# Classification of the Gut Microbiota of Patients in Intensive Care Units During Development of Sepsis and Septic Shock

**DOI:** 10.1016/j.gpb.2020.06.011

**Published:** 2021-02-17

**Authors:** Wanglin Liu, Mingyue Cheng, Jinman Li, Peng Zhang, Hang Fan, Qinghe Hu, Maozhen Han, Longxiang Su, Huaiwu He, Yigang Tong, Kang Ning, Yun Long

**Affiliations:** 1 Department of Critical Care Medicine, Peking Union Medical College Hospital, Beijing 100730, China; 2 Key Laboratory of Molecular Biophysics of the Ministry of Education, College of Life Science and Technology, Huazhong University of Science and Technology, Wuhan 430074, China; 3 State Key Laboratory of Pathogen and Biosecurity, Beijing Institute of Microbiology and Epidemiology, Beijing 100071, China; 4 Beijing Advanced Innovation Center for Soft Matter Science and Engineering (BAIC-SM), College of Life Science and Technology, Beijing University of Chemical Technology, Beijing 100029, China

**Keywords:** Sepsis, Septic shock, Gut microbiota, Enterotype, Precision medicine

## Abstract

The **gut microbiota** of intensive care unit (ICU) patients displays extreme dysbiosis associated with increased susceptibility to organ failure, **sepsis**, and **septic shock**. However, such dysbiosis is difficult to characterize owing to the high dimensional complexity of the gut microbiota. We tested whether the concept of **enterotype** can be applied to the gut microbiota of ICU patients to describe the dysbiosis. We collected 131 fecal samples from 64 ICU patients diagnosed with sepsis or septic shock and performed 16S rRNA gene sequencing to dissect their gut microbiota compositions. During the development of sepsis or septic shock and during various medical treatments, the ICU patients always exhibited two dysbiotic microbiota patterns, or ICU-enterotypes, which could not be explained by host properties such as age, sex, and body mass index, or external stressors such as infection site and antibiotic use. ICU-enterotype I (ICU E1) comprised predominantly *Bacteroides* and an unclassified genus of Enterobacteriaceae, while ICU-enterotype II (ICU E2) comprised predominantly *Enterococcus*. Among more critically ill patients with Acute Physiology and Chronic Health Evaluation II (APACHE II) scores > 18, septic shock was more likely to occur with ICU E1 (*P* = 0.041). Additionally, ICU E1 was correlated with high serum lactate levels (*P* = 0.007). Therefore, different patterns of dysbiosis were correlated with different clinical outcomes, suggesting that ICU-enterotypes should be diagnosed as independent clinical indices. Thus, the microbial-based human index classifier we propose is precise and effective for timely monitoring of ICU-enterotypes of individual patients. This work is a first step toward **precision medicine** for septic patients based on their gut microbiota profiles.

## Introduction

Patients in intensive care units (ICUs) are critically ill and exhibit inflammation and a suppressed immune system. These patients usually undergo frequent medical interventions, including administration of antibiotics, vasoactive agents, and opioids. These factors can disrupt the gut microbiota of ICU patients; such dysbiosis may increase susceptibility to hospital-acquired infections, sepsis, and multiorgan dysfunction syndrome [1], [2], [3]. Therefore, the gut microbiota should be carefully treated when performing medical interventions.

Several preliminary studies have investigated the gut microbiota of ICU patients (hereafter referred to as “ICU gut microbiota”) [4], [5], [6], [7]. ICU gut microbiota is characterized by extreme dysbiosis: a loss of health-promoting commensal microbes such as those belonging to Firmicutes and Bacteroidetes, and an increase in pathogenic microbes such as Proteobacteria [6]. However, this dysbiosis is difficult to characterize for individual patients owing to the high heterogeneity of the gut microbiota. Thus, researchers must find an accurate method of characterizing the ICU gut microbiota that is meaningful to clinical diagnosis.

Enterotype analysis [8], [9] has recently been applied in various gut microbiota studies, especially analyses of the enterotypes in healthy human guts (*i.e.*, conventional enterotypes). Interestingly, studies have revealed two patterns of conventional enterotypes [10], [11] regardless of differences in diet, genetic material, and environmental exposure. Therefore, we hypothesize that ICU patients exhibit two or more ICU gut microbiota patterns (*i.e.*, ICU-enterotypes) despite different inherited traits and external stressors such as infection type and antibiotic use.

To test our hypothesis, we recruited a cohort of ICU patients with sepsis or septic shock and investigated their gut microbiota compositions. Two ICU-enterotype patterns were common during the 9-day sampling, despite the subjects’ heterogeneous infection types and antibiotic use. Patients with septic shock were more likely to present ICU-enterotype I (ICU E1), which also was correlated with high serum lactate levels. These results might be due to the microbiota patterns of ICU E1, which included *Bacteroides* and a dominant unclassified genus from Enterobacteriaceae; ICU-enterotype II (ICU E2), on the other hand, contained predominantly *Enterococcus*. Additionally, the microbial-based human index (MHI) classifier proposed in this study facilitates timely monitoring of patients’ ICU-enterotypes for microbiome-based precision medicine.

## Results

A cohort of 64 ICU patients (aged 57.71 ± 18.85 years) who developed sepsis or septic shock were enrolled in this study. Thirty-two patients received carbapenem with or without combinations of other antibiotics (the carbapenem-use group), and 30 patients received other antibiotics such as cephalosporins, penicillin, azithromycin, fluoroquinolones, or vancomycin (the non-carbapenem-use group). Patients who received carbapenem were separated because of the broad-spectrum antibacterial activity and the wide usage of carbapenem in critically ill patients [12], [13]. The remaining two patients used no antibiotics during the sample collection period. We collected 131 fecal samples from these 64 patients during their first 9 days in the ICU for ICU-enterotype identification. To avoid repeated measures from the same individual, we used their first fecal samples (*n* = 64) and the corresponding clinical information, including Sequential Organ Failure Assessment (SOFA) score (10.67 ± 4.02), Acute Physiology and Chronic Health Evaluation (APACHE) II score (20.37 ± 8.14), and lactate level (2.33 ± 2.88 mM), to investigate the correlation between microbiome compositions and clinical parameters. Table S1 lists the antibiotics used and detailed clinical information for the 64 patients on the day of collection of their first fecal samples. Figure S1 and Table S2 describe the distribution and characteristics of the 131 fecal samples obtained over the 9-day period.

### Two ICU-enterotypes were identified in the ICU gut microbiota

**Figure 1:**
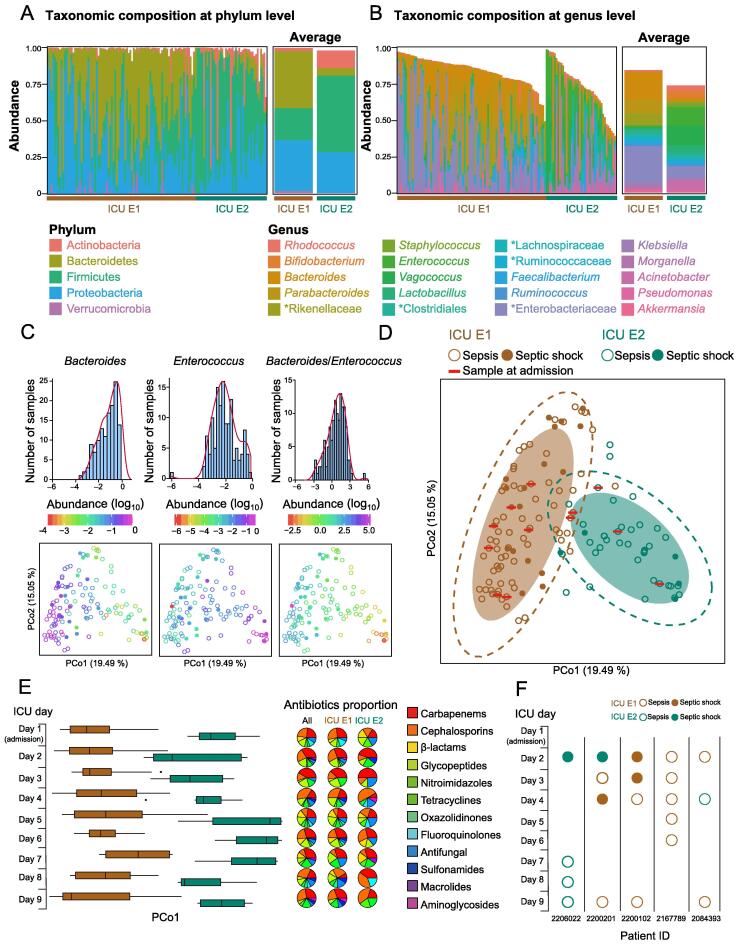
**Two ICU-enterotypes identified in ICU patients with sepsisorseptic shock** Taxonomic composition of 131 fecal samples at the phylum (**A**) and genus (**B**) levels. Taxa with relative abundances > 1% among all samples are plotted. Unclassified genera are designated as a higher rank marked by asterisks. The left panel represents the taxonomic composition of each sample; the right panel represents the taxonomic composition of the two ICU-enterotypes using the mean abundance calculated from the data in the left panel. Brown and green bars represent the ICU-enterotypes to which these samples belong. **C.** Distributions of the log-transformed (log_10_) relative abundances of the most significantly differing genera. The top panel displays the observed distributions using a frequency distribution histogram with density curve. The bottom panel displays these distributions in ICU-enterotype space represented by JSD-based PCoA plot. Solid circles represent samples from septic shock patients; hollow circles represent samples from septic patients. Colored PCoA plot: log-transformed (log_10_) relative abundances of the genera for each sample. **D.** Gut microbiota compositions of individual patients with ICU E1 (*n* = 89) and ICU E2 (*n* = 42) are plotted on a JSD-based PCoA plot at the genus level. Shaded ellipses represent the 80% CI; dotted ellipses represent the 95% CI. **E.** In the left panel, the daily fecal sample distributions are plotted against the PCo1 axis (D). Boxes represent the IQR between the first and third quartiles; the line inside represents the median. Whiskers denote the lowest and highest values within 1.5× IQR from the first and third quartiles, respectively. In the right panel, the proportions of antibiotics used on each day are displayed using all samples, ICU E1 samples, and ICU E2 samples, respectively. **F.** Variation in ICU-enterotypes from five patients (IDs shown at the bottom) along the 9-day observation. Figure S1 shows all patients’ results. JSD-based PCoA, Jensen-Shannon distance-based principal coordinate analysis; ICU E1, ICU-enterotype I; ICU E2, ICU-enterotype II; CI, confidence interval; IQR, interquartile range.

Two ICU-enterotypes were identified using previously reported methods [9] and statistical analysis (Table S3). The two ICU-enterotypes differed significantly in their taxonomic distributions at the phylum, class, order, family, and genus levels (Table S4). The microbial richness [quantified by the number of observed operational taxonomic units (OTUs)] was lower in samples of ICU E1 than in samples of ICU E2 (*P* = 2.064E−4, Mann-Whitney-Wilcoxon Test), while the microbial diversity (Shannon index) did not significantly differ (*P* = 0.057). At the phylum level, Bacteroidetes was more prevalent in ICU E1 (*P* = 3.086E−14), while Actinobacteria and Firmicutes were more prevalent in ICU E2 (*P* = 6.20E−10 and *P* = 9.25E−08, respectively, Kruskal-Wallis test; Figure 1A). At the genus level, *Bacteroides* and *Parabacteroides* were more prevalent in ICU E1 (*P* = 6.650E−12 and *P* = 1.481E−11, respectively), while *Enterococcus*, *Vagococcus*, and *Lactobacillus* were more prevalent in ICU E2 (*P* = 7.516E−13, *P* = 2.144E−11, and *P* = 0.002, respectively, Kruskal-Wallis test; Figure 1B). Interestingly, an unclassified genus of Enterobacteriaceae was dominant in ICU E1 (0.251 ± 0.026 in ICU E1, 0.082 ± 0.016 in ICU E2; *P* = 9.49E−06; Figure 1B).

*Bacteroides* (dominant in ICU E1), *Enterococcus* (dominant in ICU E2), and the *Bacteroides*/*Enterococcus* ratio showed obvious gradient distributions against the PCo1 axis (Figure 1C). The distributions of both *Bacteroides* and *Enterococcus* were near log-normal, suggesting that *Bacteroides* was enriched in ICU E1, and *Enterococcus* was enriched in ICU E2. These results indicate that, unlike the conventional enterotypes characterized by *Bacteroides* and *Prevotella* gradient distributions [8], ICU-enterotypes largely contain *Bacteroides* and *Enterococcus* gradient distributions. Moreover, the gradient distribution of the unclassified genus from Enterobacteriaceae was observed against the PCo2 axis (Figure S2).

The pathogens identified from positive bacterial cultures of the infection sites partially overlapped with the gut microbiome profiles at the genus level (Tables S1 and S4). Most of the overlaping genera, such as *Enterococcus*, *Staphylococcus*, *Corynebacterium*, and *Acinetobacter*, were differentially distributed between the two ICU-enterotypes, confirming the associations between the gut microbiome and extraintestinal causative pathogens.

### ICU-enterotypes were pervasive in the ICU patient cohort

The two identified ICU-enterotypes were pervasive in the cohort of ICU patients with sepsis or septic shock. The samples taken at admission mixed with all samples collected during the ICU stay showed no clustering pattern (Figure 1D) and were distributed among the two ICU-enterotypes. We then displayed the distributions of all 131 samples on each of the 9 days to test whether the enterotypes remained when using one sample from each patient (Figure 1E). Interestingly, the ICU-enterotypes were obvious on each day, despite the differences in subject sets and daily antibiotic category proportions. Furthermore, the ICU-enterotypes did not significantly differ in distribution of carbapenem use before and after ICU admission (Figure S3A and B; Mann-Whitney-Wilcoxon test). The infection site distribution was correlated with ICU-enterotypes; samples from patients with nonpulmonary infections were enriched with ICU E1 (Figure S3C). However, this correlation requires further validation on a larger scale. Antibiotics and infection types can affect the ICU gut microbiota; however, they did not determine the ICU-enterotypes. The ICU-enterotypes may result from the patterns of dysbiosis in the ICU gut microbiota owing to factors such as inherited traits, medical treatments, and infection types.

**Figure 2:**
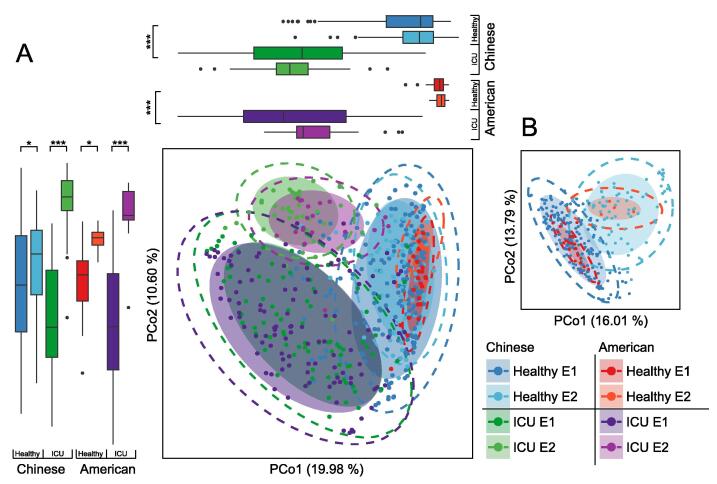
**ICU-enterotypes in Chinese-ICU and American-ICU patients are similar and largely differ from healthy enterotypes** **A.** The central PCoA displays the gut microbiota compositions of Chinese ICU E1 (*n* = 89) and Chinese ICU E2 (*n* = 42), Chinese healthy E1 (*n* = 188) and Chinese healthy E2 (*n* = 76), American ICU E1 (*n* = 108) and American ICU E2 (*n* = 21), and American healthy E1 (*n* = 22) and American healthy E2 (*n* = 4). Box plots show sample distributions of these groups against the PCo1 and PCo2 axes. Boxes represent the IQR between first and third quartiles; the line inside represents the median. Whiskers denote the lowest and highest values within 1.5× IQR from the first and third quartiles, respectively. Statistical significance was tested using the Mann-Whitney-Wilcoxon test (*, *P* < 0.05; ***, *P* < 0.001). **B.** The PCoA only displays the gut microbiota compositions of Chinese healthy E1 and E2, and American healthy E1 and E2. In the PCoA plots, shaded ellipses represent the 80% CI; dotted ellipses represent the 95% CI.

ICU-enterotypes were also found in a cohort of 129 American ICU patients [6]. We performed the same enterotype analysis on this cohort and found two American ICU-enterotypes driven by *Bacteroides* and *Enterococcus*, which were also recognized as driving the Chinese ICU-enterotypes. *Bacteroides* was more prevalent in American ICU E1 (*P* < 2.2E−16, Mann-Whitney-Wilcoxon test; 0.206 ± 0.014) than in American ICU E2 (0.024 ± 0.007), whereas *Enterococcus* was more prevalent in American ICU E2 (*P* < 2.2E−16, Mann-Whitney-Wilcoxon test; 0.180 ± 0.033) than in American ICU E1 (0.010 ± 0.002). We subsequently performed enterotype analysis on two healthy cohorts comprised of healthy Chinese [14] and healthy American [15] subjects to compare the ICU-enterotypes. The American ICU-enterotypes overlapped with the Chinese ICU-enterotypes in the principal coordinate analysis (PCoA) plot, and these four ICU-enterotypes were separated from the four healthy enterotypes against the PCo1 axis (*P* < 2.2E−16, Mann-Whitney-Wilcoxon test; Figure 2A). Notably, owing to the large differences between ICU microbiota and healthy microbiota, the samples of healthy enterotypes I and II (Healthy E1 and E2) were clustered when we projected these eight enterotypes on the same PCoA plot. Removing the ICU samples from the PCoA revealed the separation between the healthy enterotypes (Figure 2B). Thus, the ICU-enterotypes in both Chinese and American patients had similar taxonomic compositions and largely differed from healthy enterotypes.

The stability of ICU-enterotypes of Chinese ICU patients during the first 9 days remains unknown owing to the lack of samples at several time points. Fourteen patients exhibited ICU-enterotype variation (Figure S1), while the remaining patients had no change in ICU-enterotype, likely owing to the small sample size. Notably, one patient (ID 2167789) had a stable ICU-enterotype, even over a continuous sampling of 5 days (Figure 1F). ICU-enterotypes might be less stable than conventional enterotypes because ICU patients are frequently exposed to various external stressors that can greatly affect the gut microbiota. Considering the relative instability of ICU-enterotypes and their correlation with septic shock, patients’ ICU-enterotypes should be adequately monitored.

### A highly effective binary classifier for discriminating ICU-enterotypes

**Figure 3:**
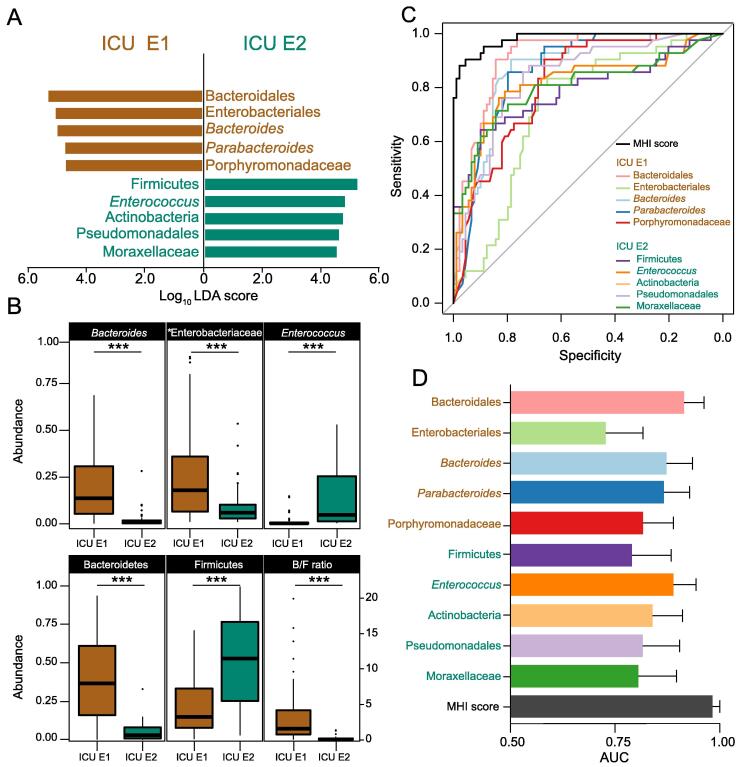
**MHI scores show greater discriminant ability thansingle taxonomic biomarkers in ICU-enterotypeclassification** **A.** Ten taxonomic biomarkers selected by LEfSe and mRMR are shown with the length of the bar representing the LDA score (log_10_) and the color indicating which ICU-enterotypes these biomarkers belong to. **B.** The relative abundances of the most dominant biomarkers selected only by LEfSe at the phylum and genus levels differ largely between the ICU-enterotypes. Boxes represent the IQR between the first and third quartiles; the line inside represents the median. Whiskers denote the lowest and highest values within 1.5× IQR from the first and third quartiles, respectively. Statistical significance was tested using the Mann-Whitney-Wilcoxon test (***, *P* < 0.001; n.s., not significant). **C.** ROC was performed for all 131 samples using the MHI score and the 10 individual taxonomic biomarkers. Figure S4 displays the ROCs with 95% CI of each biomarker or MHI score. **D.** AUC with 95% CI calculated from the results of (C). LDA, linear discriminate analysis; LEfSe, LDA effect size; mRMR, minimum redundancy maximum relevance feature selection; ROC, receiver operating characteristic; MHI, microbial-based human index; AUC, area under the curve.

Identifying the ICU-enterotype of a single sample can facilitate timely monitoring of ICU-enterotypes. Thus, we proposed a binary classifier based on the nonredundant taxonomic biomarkers of the ICU-enterotypes selected by linear discriminate analysis (LDA) effect size (LEfSe) and minimum redundancy maximum relevance feature selection (mRMR). Ten selected taxonomic biomarkers at different phylogenetic levels showed great discriminant ability, with Log_10_ LDA score > 4.0 (**Figure 3**A). In addition, among all the biomarkers identified only by LEfSe，the phyla Bacteroidetes and Firmicutes and the genera *Bacteroides* and *Enterococcus* were the most discriminant biomarkers at the phylum and genus levels, respectively, which showed large differences in abundance proportions between the two ICU-enterotypes (Figure 3B).

We then combined these ten biomarkers to produce MHI scores for each fecal sample as described in the Metarials and methods section. We first compared the ability of the MHI score to classify all 131 samples into two ICU-enterotypes with that of the ten individual taxonomic biomarkers (Figure 3C and D, Figure S4). The MHI score improved the effectiveness of classifying these samples as ICU E1 or II, with the highest area under the curve (AUC) being 0.9791 (95% CI: 0.9586–0.9997) compared with that of any individual taxonomic biomarker.

We then proposed an MHI score threshold as the judgment criterion for the MHI classifier for clinical diagnosis. The threshold was trained to 1.017 in the training set of 106 Chinese ICU samples, which was applied to the testing set I containing 25 Chinese ICU samples. To confirm the practicality of this classifier, we applied it to the testing set II, consisting of 129 American ICU samples, using the same set of biomarkers to calculate the MHI score with the same trained threshold (1.017). The MHI classifier with the trained threshold (1.017) yielded an AUC value of 0.982 and an F1 score of 0.929 during the training (Table 1). This classifier was effective for classification during the testing process, with an AUC value of 0.985 and an F1 score of 0.903 in the testing set I and an AUC value of 0.884 and an F1 score of 0.928 in the testing set II. Therefore, the MHI classifier is applicable and highly effective in both ICU cohorts and may serve as an effective classification strategy for assessing ICU-enterotypes in ICU clinical trials.

**Table 1:** **Performance evaluation ofMHI for classifying ICU-enterotypes**

*Note*: Samples of the training set and testing I set are from Chinese ICU patients, and samples of the testing II set are from American ICU patients [6]. MHI, microbial-based human index.

### Two ICU-enterotypes were correlated with septic shock and serum lactate

#### Correlation with septic shock

The ICU-enterotypes were correlated with septic shock. The samples from septic patients (106 samples from 46 patients) were highly prevalent in both ICU E1 (69 samples from 33 patients, 65.1%) and ICU E2 (37 samples from 13 patients, 34.9%; Figure 1D and Table 2). However, the samples from patients with septic shock (25 samples from 18 patients) were mostly classified as ICU E1 (20 samples from 14 patients, 80%), with five samples from four patients (20%) classified as ICU E2. Because the microbiome compositions and clinical conditions (*e.g.*, the status of sepsis or septic shock and APACHE II score) of each sample varied daily under clinical intervention, we viewed these samples as independent. Higher APACHE II scores indicated more severe disease conditions [16], [17], [18], and patients who died had a tendency of higher APACHE II scores than those who survived (median ± interquartile range: 22.5 ± 14 *vs.* 18.5 ± 11, *P* = 0.1, Mann-Whitney-Wilcoxon test). We thus set a cutoff value for APACHE II scores as > 18 to determine whether the samples were from patients with severe disease conditions. Among samples with APACHE II scores > 18 (83 of 131), a significantly larger proportion (86.7% *vs.* 57.4%, *P* = 0.041, Fisher's exact test) of samples from septic shock patients presented ICU E1 than did samples from septic patients. Samples with APACHE II scores ≤ 18 did not differ significantly (*P* = 0.675) between these two patient groups. Thus, patients with septic shock were more likely to present ICU E1, especially those with a more critical status.

**Table 2:** **ICU-enterotype distributionof patients with sepsis or septic shock**

*Note*: *P* values were calculated using Fisher's exact test. APACHE II, Acute Physiology and Chronic Health Evaluation II; ICU E1, ICU-enterotype I; ICU E2, ICU-enterotype II.

#### Correlation with serum lactate

**Figure 4:**
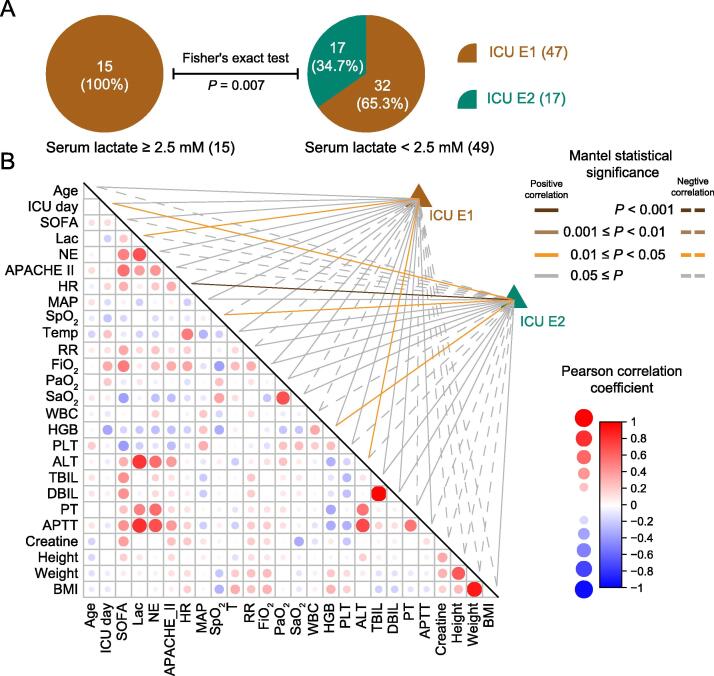
**Two ICU-enterotypes are correlated with different clinical parameters** **A.** The pie charts show proportions of patients with each ICU-enterotype in the high-lactate (left panel) and low-lactate (right panel) groups. Fisher’s exact test was used to evaluate the statistical significance of the difference in these proportions. **B.** The correlation matrix was produced using Pearson correlation coefficients between each pair of Z-score-transformed clinical parameters. Circle size and color refer to the strength of the Pearson correlation coefficients, as shown on the right of the matrix. Correlations between taxonomic compositions of ICU-enterotypes and each Z-score-transformed clinical parameter were calculated using the Mantel test. Samples of ICU EI and ICU EII were tested. Line color refers to the Mantel statistical significance. The solid line indicates a positive correlation; the dashed line indicates a negative correlation. Table S6 lists the full names of the clinical parameters and detailed values of the Mantel test.

To look for correlations between ICU-enterotypes and clinical parameters, we used a subset of 64 samples (one per patient) to eliminate the influence of heterogeneity on patient sample size. These 64 samples were composed of the first collected sample of each patient, whose enterotypes were designated according to enterotype analysis. Additionally, the clinical parameters were recorded on the same day as the fecal sample collection (Table S1). The distributions of sex (*P* = 0.4), age (*P* = 0.68), sample collection day (*P* = 0.7323), carbapenem use (*P* = 0.57), and breathing support (*P* = 0.73) did not significantly differ between the two ICU-enterotypes, indicating that no sample selection bias existed between the two ICU-enterotypes (Table S5). We then tested whether crucial clinical parameters for evaluating the disease condition severity of septic patients differed between the two ICU-enterotypes. SOFA scores (*P* = 0.8), APACHE II scores (*P* = 0.474), and 28-day survival rates (*P* = 0.36) did not significantly differ between the two ICU-enterotypes (Table S5). Interestingly, patients with ICU E1 had a tendency of higher serum lactate levels than patients with ICU E2 (2.66 ± 3.30 *vs.* 1.42 ± 0.52, *P* = 0.07). We then divided the patients into the high-lactate (serum lactate ≥ 2.5 mM) and low-lactate (serum lactate < 2.5 mM) groups. All patients (100%) in the high-lactate group presented ICU E1, while 34.7% of patients in the low-lactate group presented ICU E2 (*P* = 0.007; Figure 4A). ICU E1 was correlated with high serum lactate levels.

We subsequently performed a Mantel test [19] on these 64 samples to test whether patients’ clinical parameters could explain the gut microbiota variation within ICU-enterotypes. The clinical parameters were correlated and could thus indicate the illness severity (Figure 4B). Only a few clinical parameters could explain the gut microbiota variation (Figure 4B; Table S6). The gut microbiota variation of ICU E1 was positively correlated with serum lactate concentrations (*r* = 0.148, *P* = 0.0481), suggesting that patients with ICU E1 had more diversified gut microbiota patterns if they also had differing serum lactate levels. However, this correlation was not observed in patients with ICU E2 (*r* = −0.079, *P* = 0.815).

## Discussion

During the development of sepsis or septic shock, as well as various medical treatments, ICU patients exhibited two distinct gut microbiota patterns, designated as ICU-enterotypes. Individual host properties, such as age, sex, and body mass index, as well as external stressors, such as infection site and antibiotic use, could not explain the ICU-enterotypes. Although the causes of ICU-enterotypes remain unknown, their prevalence and correlation with septic shock and serum lactate levels make them pragmatic independent clinical parameters of the gut microbiota for clinical trials.

ICU gut microbiota has been previously described as displaying extreme dysbiosis, with a loss of health-promoting bacteria and a growth of pathogenic bacteria. However, the two ICU-enterotypes identified here indicate that this dysbiosis contains two distinct patterns with corresponding sets of bacterial loss or growth. For example, ICU E2 exhibited a loss of *Bacteroides* but a growth of *Enterococcus*, while ICU E1 presented the opposite result. Moreover, the bacteria were differentially distributed between the two ICU-enterotypes and partially overlapped with the extraintestinal causative pathogens at the genus level. Furthermore, similar taxonomic characteristics of ICU-enterotypes were also found in an American ICU patient cohort [6]. Therefore, dysbiosis of the ICU gut microbiota should be analyzed and treated differently according to ICU-enterotypes.

Despite both ICU-enterotypes exhibiting extreme dysbiosis, their different microbiota patterns were correlated with different outcomes. For example, the larger Bacteroidetes/Firmicutes ratio in ICU E1, which more than 10 has been correlated with a higher death rate in ICU patients [5], may reflect a critical status among patients. In addition, an unclassified genus of Enterobacteriaceae was dominant in ICU E1. Because another member of this family, *Enterobacter*, can cause various nosocomial infections, such as neonatal sepsis with meningitis [20], the identity and potential pathogenicity of this unclassified genus should be determined. Additionally, *Enterococcus*, which can cause urinary tract infections, abdominal-pelvic infections, and endocarditis [21], [22], [23], was dominant in ICU E2. *Enterococcus* has also been reported to enhance immunity and inhibit overgrowth of opportunistic pathogens by producing short-chain fatty acids [24], [25] and bacteriocins [26], [27], [28]. Therefore, the enterococcal overgrowth in ICU E2 might be associated with a lower occurrence of septic shock in patients with ICU E2. However, more studies are needed to test this speculation.

Although both ICU-enterotypes may lead to severe pathogenic diseases, the different clinical outcomes are linked to different patterns of dysbiosis of the ICU-enterotypes. For example, there exists a higher correlation between ICU E1 and septic shock. Considering this link, the MHI classifier proposed herein may facilitate timely monitoring of ICU-enterotype variations among patients. The MHI classifier emphasizes the improved classification ability by combining multiple biomarkers in comparison to using just a single biomarker. We believe that this strategy can improve the microbiome classification of the other cohort.

ICU-enterotypes can serve as both independent clinical parameters for characterizing ICU gut microbiota and indicators of disease severity. Our samples from severe disease conditions showed that ICU E1 was correlated with septic shock. Additionally, ICU E1 was correlated with high serum lactate levels, suggesting a deterioration in critical hemodynamics. Elevated serum lactate concentration reflects increased anaerobic glycolysis and is commonly considered a marker of tissue hypoxia or hypoperfusion in critically ill patients [29], [30], [31], [32]. Studies have suggested that serum lactate concentration > 2 mM was independently correlated with mortality in both shock and nonshock patients [32]. Other studies argue that blood lactate concentration > 0.75 mM was independently correlated with increased mortality [33]. Despite disputes over the cutoff value, higher lactate levels indicate a greater risk of death, and treatments guided by lactate levels have successfully decreased mortality rates [34]. Therefore, ICU-enterotypes may be markers of disease severity, which would guide clinical interventions.

This study has some limitations. First, the lack of fecal samples at several time points prevented determining the evolution of ICU-enterotypes in individual patients and investigating the predictive ability of ICU-enterotypes for developing sepsis or septic shock. Second, the relatively small cohort in our study may have limited the statistical power of the results. Finally, the patients’ comorbidities were not considered because of their heterogeneity. Nevertheless, all factors, such as disease variety and medical treatments, can cause dysbiosis. The effects of these factors on the gut microbiota may differ; however, they all cooperatively resulted in two ICU-enterotypes. This study revealed the existence of these two ICU-enterotypes among a cohort of ICU patients with sepsis or septic shock.

## Conclusion

Despite ICU gut microbiota being of different dysbiosis, two patterns of dysbiosis, designated as ICU-enterotypes, were predominant among the ICU patient cohort. ICU E1 reflected a more severe septic shock status and was correlated with deterioration of critical hemodynamics, suggesting higher levels of critical treatment. Patients with different ICU-enterotypes should be treated with different strategies. Furthermore, observation of the ICU-enterotypes in both the Chinese and American ICU cohorts might suggest that ICU-enterotypes are universal among global ICU populations. A better understanding of these ICU-enterotypes can contribute to more general practical guidelines in ICU clinical treatment.

## Materials and methods

### Subjects and clinical assessments

We recruited four sets of microbiome samples from a Chinese ICU cohort with 131 samples, a healthy Chinese cohort with 264 samples [14], an American ICU cohort with 129 samples [6], and a healthy American cohort with 26 samples [15]. For Chinese ICU patients, the study was conducted in the Critical Care Department of Peking Union Medical College Hospital (PUMCH) of China, a tertiary referral hospital with 30 beds in the Critical Care Department. We enrolled ICU patients with sepsis or septic shock who were either directly admitted to the ICU or transferred to the ICU from a hospital ward from February 2016 to February 2017. SOFA and APACHE II scores were calculated daily. Sepsis was defined as when a patient was treated with systemic therapeutic antibiotics for a suspected infection, accompanied by an increased SOFA score of ≥ 2 points [35]. Septic shock was identified by a vasopressor requirement of maintaining a mean arterial pressure of ≥ 65 mmHg and a serum lactate level > 2 mM (>18 mg/dl) in the absence of hypovolemia [35]. Other clinical assessments, including physiological parameters, serum lactate levels, and blood test results, were obtained daily for each patient. Antibiotic use, infection site, pathogens from positive bacterial cultures, ICU stay duration, mechanical ventilation, and 28-day survival rate were also recorded for each patient.

### Fecal sample collection and 16S rRNA gene sequencing

The included patients were observed for their first 9 consecutive days in the ICU (starting from the day of admission). Antibiotics were administered to ICU patients immediately upon hospital admission and before the fecal sample collection on each of the 9 days, including the admission day. Fresh fecal samples were collected from each patient via defecation. Enemas were used for patients with constipation and were performed by nurses using a glycerin enema as per the instructions given by the intensivists. If patients produced multiple fecal specimens on the same day, only one was collected for the test. If patients could not produce a fecal specimen on any day of the observation, no fecal sample was available for those days. Fresh fecal samples were immediately frozen at −80 °C. Total bacterial DNA was subsequently extracted from homogenized fecal samples using the QIAamp® Fast DNA Stool Mini kit (Catalog No. 51604, QIAGEN, Hilden, Germany) as per the manufacturer’s protocol without modifications. The V3/V4 hypervariable regions of the 16S rRNA gene were sequenced using the Illumina MiSeq platform. Mothur [36] was used to merge paired-end reads and to trim the reads to meet the following quality standards: a quality Phred score ≥ Q20, no ambiguous bases, homo-polymers shorter than 8 bp, and a read length of 300–500 bp. The trimmed reads were then aligned against SILVA 132 reference files to identify chimeras (Silva-based alignment of template file for chimera; Slayer, release 132). Identified chimeras were then removed using the UCHIME algorithm [37]. The remaining high-quality sequences were clustered into OTUs at a 97% sequence identity threshold using UCLUST [38]. The most abundant reads from each OTU cluster were taxonomically identified using RDP classifier [39]; only annotations with ≥ 80% confidence levels were accepted. Rarefaction was set at 4000 reads based on curve plateaus for alpha diversity. The 16S rRNA gene sequencing data of the ICU American cohort [6], the healthy Chinese cohort [14], and the healthy American cohort [15] are available in the Sequence Read Archive (SRA) at the National Center for Biotechnology Information (SRA: ERP012810, SRP107602, and ERP001500, respectively), and are available at https://trace.ncbi.nlm.nih.gov/Traces/sra/?study=ERP012810, https://trace.ncbi.nlm.nih.gov/Traces/sra/?study=SRP107602, and https://trace.ncbi.nlm.nih.gov/Traces/sra/?study=ERP001500, respectively.

### Identification of ICU-enterotypes

Relative abundances of OTUs up to the genus level for each sample were used to analyze the enterotypes [9]. First, the Jensen-Shannon distance (JSD) between each sample was calculated to produce a JSD matrix using relative abundances at the genus level. Second, the partitioning around medoids (PAM) clustering algorithm was performed on this distance matrix to cluster samples; the Calinski-Harabasz (CH) index [40] was used to assess the optimal number of clusters. Third, the silhouette coefficient (SI) [41] was calculated to evaluate the statistical significance of the clustering. We set and evaluated the number of clusters from zero to ten, and we found that two clusters, ICU E1 and ICU E2, were of the highest CH and SI. The PAM clustering algorithm and calculation of the CH and SI indices are available in the R packages ‘cluster’ (version 2.0.7) and ‘clusterSim’ (version 0.47-1).

The two identified ICU-enterotypes were visualized using PCoA in the R packages ‘ade4’ (version 1.7-11) and ‘ggplot2’ (version 2.2.1). All fecal samples were projected onto the two-dimensional plane using PCo1 and PCo2 axes as the most discriminant axes. Shaded ellipses represented the 80% CI, and dotted ellipses indicated the 95% CI, thus wrapping the samples within an enterotype.

### Detection of taxonomic biomarkers of ICU-enterotypes

Taxonomic biomarkers of the two identified ICU-enterotypes were first picked from the taxonomic abundance tables using LEfSe [42]. The selected biomarkers with Log_10_ LDA score > 2.0 were then used as the inputs for mRMR [43] to determine five nonredundant taxonomic biomarkers for each ICU-enterotype.

### MHI classifier for discriminating ICU-enterotypes

The MHI was used to determine the combined effects of the taxonomic biomarkers for discriminating ICU-enterotypes. For each sample, the ten taxonomic biomarkers selected by mRMR were organized according to the formula below to produce the MHI:

$$MHI\;score=\frac{\sum_{i=1}^{n}ABU_{r}\left(S_{i}\right)}{\sum_{j=1}^{m}ABU_{r}\left(S_{j}\right)}$$

The numerator was the sum of the relative abundances (*ABU_r_*) of five selected taxonomic biomarkers (*S_i_*) of ICU E1. The denominator was the sum of the relative abundances of five selected biomarkers (*S_j_*) of ICU E2. A ROC curve with 95% CI and an AUC value with 95% CI were generated for the 131 samples using 9999 stratified bootstrap replicates to compare the classification ability of the ten individual taxonomic biomarkers and the MHI score.

An MHI score threshold was then proposed as the judgment criterion for classifying ICU-enterotypes, *i.e.*, samples with MHI scores above this threshold were classified as ICU E1, and samples with MHI scores below this threshold were classified as ICU E2. To train and test the MHI classifier, 131 samples of the Chinese ICU cohort were randomly divided into the training set (106 samples, 80%) and the testing set I (25 samples, 20%). During training, the threshold was initially set at the min MHI score among training set samples, then increased by the value of (max MHI score − min MHI score)/1000 at every step. This process was terminated when sensitivity and specificity of the classification reached the local optimal values. During testing, the trained threshold was first used as the judgment criterion to classify the testing set I. To further confirm the practicality of the trained MHI classifier, another ICU cohort consisting of 129 American ICU samples [6] served as the testing set II in the testing process. The MHI score of the testing set II was calculated using the same set of biomarkers as in the Chinese ICU cohort, and the testing set II was classified using the same trained MHI threshold as described above.

## Ethical statement

Written informed consent was obtained from all subjects or their legal representatives. Ethical approval was received from the Medical Ethics Committee of PUMCH (ethical reference number HS-1350), and all experiments were conducted in accordance with the declaration of Helsinki.

## Code availability

The codes for selecting taxonomic biomarkers and building the MHI classifier are available in GitHub at https://github.com/HUST-NingKang-Lab/MH-E-I.

## Data availability

The 16S rRNA gene sequencing data of the ICU Chinese cohort are available in the Genome Sequence Archive [44] at the National Genomics Data Center, Beijing Institute of Genomics, Chinese Academy of Sciences / China National Center for Bioinformation (GSA: CRA002354), and are publicly accessible at https://bigd.big.ac.cn/gsa/browse/CRA002354.

## CRediT author statement

**Wanglin Liu:** Methodology, Formal analysis, Investigation, Resources, Data curation, Writing - original draft, Writing - review & editing. **Mingyue Cheng:** Methodology, Software, Validation, Formal analysis, Data curation, Writing - original draft, Writing - review & editing, Visualization. **Jinman Li:** Formal analysis, Investigation, Resources, Data curation. **Peng Zhang:** Software, Formal analysis. **Hang Fan:** Investigation, Resources. **Qinghe Hu:** Investigation, Resources. **Maozhen Han:** Software, Formal analysis. **Longxiang Su:** Investigation, Resources. **Huaiwu He:** Investigation, Resources. **Yigang Tong:** Conceptualization, Writing - review & editing, Supervision. **Kang Ning:** Conceptualization, Writing - review & editing, Supervision, Funding acquisition. **Yun Long:** Conceptualization, Writing - review & editing, Supervision, Project administration, Funding acquisition. All authors read and approved the final manuscript.

## Competing interests

The authors have declared no competing interests.

## Supplementary material

Supplementary Figure S1 **Distributions of 131 fecal samples from 64 ICU patients during their first 9 days in ICU** Each row represents a patient; each column represents the day. Day 1 refers to the admission day. The last column represents the 28-day mortality. Only fecal samples designated by colored circles were obtained and analyzed to calculate enterotypes. Circles represent the daily characteristics of each patient; color represents the ICU-enterotypes, and filling (filled or not) represents sepsis or septic shock. ICU E1, ICU-enterotype I. ICU E2, ICU-enterotype II.

Supplementary Figure S2 **The distribution of the log-transformed relative abundance of the unclassified genus of Enterobacteriaceae in the two ICU-enterotypes** The left panel displays the distribution using a frequency distribution histogram with a density curve. The right panel shows the distribution in ICU-enterotype space. Solid circles represent samples from patients with septic shock; hollow circles represent samples from septic patients. The unclassified genus of Enterobacteriaceae, which was dominant in ICU E1, showed obvious gradient distributions against the PCo 2 axis.

Supplementary Figure S3 **Distributions of antibiotic use before/after ICU admission****and infection sites for samples in ICU-enterotype space** The circle color and shape indicate the ICU-enterotypes and characteristics, including antibiotic type (A and B) and infection site (C). In all plots, shaded ellipses represent the 80% CI; dotted ellipses represent the 95% CI. Boxes represent the IQR between the first and third quartiles; the line inside represents the median. Whiskers denote the lowest and highest values within 1.5× IQR from the first and third quartiles, respectively. Statistical significance was tested using the Mann-Whitney-Wilcoxon test (***, *P* < 0.001; n.s., not significant). CI, confidence interval; IQR, interquartile range.

Supplementary Figure S4 **Comparison of the ability to classify ICU-enterotypes between the ten taxonomic biomarkers and the MHI score** The ROC curve with 95% CI and AUC with 95% CI were generated for the 131 fecal samples using 9999 stratified bootstrap replicates.

Supplementary Table S1 **The clinical parameters****of 64 ICU patients**

Supplementary Table S2 **The characteristics****of 131 fecal samples**

Supplementary Table S3 **The statistical evaluation of the number****of clusters, based on JSD distance matrix at the genus level using 131 fecal samples**

Supplementary Table S4 **The comparisons of distributions of****taxonomic composition between two ICU-enterotypes**

Supplementary Table S5 **The comparisons of phenotypic characteristics****of 64 patients between two ICU-enterotypes**

Supplementary Table S6 **The correlations between taxonomic composition****of first fecal samples of 64 patients and their corresponding clinical parameters**
