# Supplementary figures and images for: Classification of the Gut Microbiota of Patients in Intensive Care Units During Development of Sepsis and Septic Shock
Source: Genomics Proteomics Bioinformatics. 2021 Feb 17;18(6):696–707. doi: 10.1016/j.gpb.2020.06.011 (PMC8377022; doi:10.1016/j.gpb.2020.06.011)

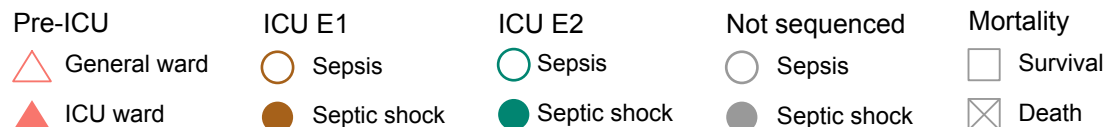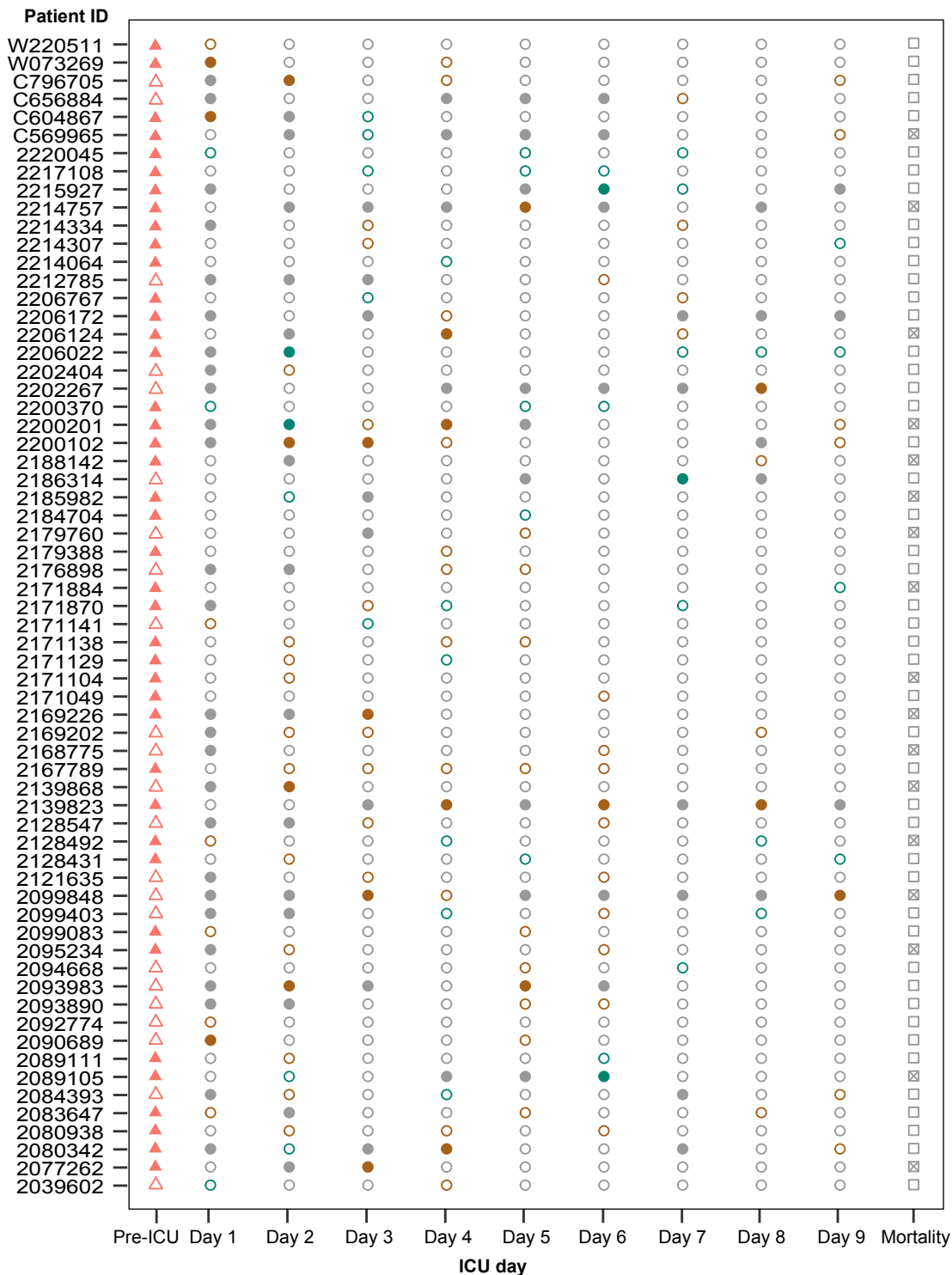

Supplement: Supplementary Figure S1 — Distributions of 131 fecal samples from 64 ICU patients during their first 9 days in ICU Each row represents a patient; each column represents the day. Day 1 refers to the admission day. The last column represents the 28-day mortality. Only fecal samples designated by colored circles were obtained and analyzed to calculate enterotypes. Circles represent the daily characteristics of each patient; color represents the ICU-enterotypes, and filling (filled or not) represents sepsis or septic shock. ICU E1, ICU-enterotype I. ICU E2, ICU-enterotype II. [file mmc1.pdf]

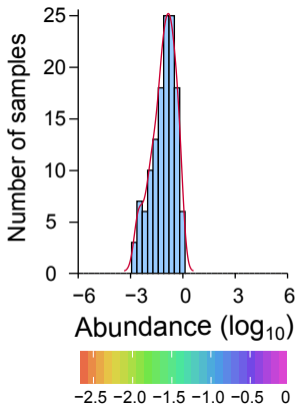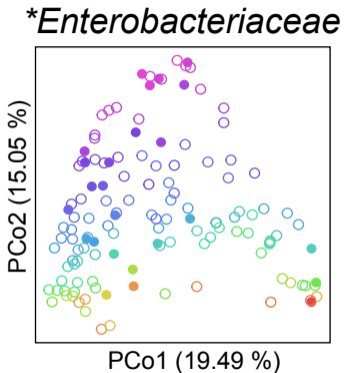

Supplement: Supplementary Figure S2 — The distribution of the log-transformed relative abundance of the unclassified genus of Enterobacteriaceae in the two ICU-enterotypes The left panel displays the distribution using a frequency distribution histogram with a density curve. The right panel shows the distribution in ICU-enterotype space. Solid circles represent samples from patients with septic shock; hollow circles represent samples from septic patients. The unclassified genus of Enterobacteriaceae, which was dominant in ICU E1, showed obvious gradient distributions against the PCo 2 axis. [file mmc2.pdf]

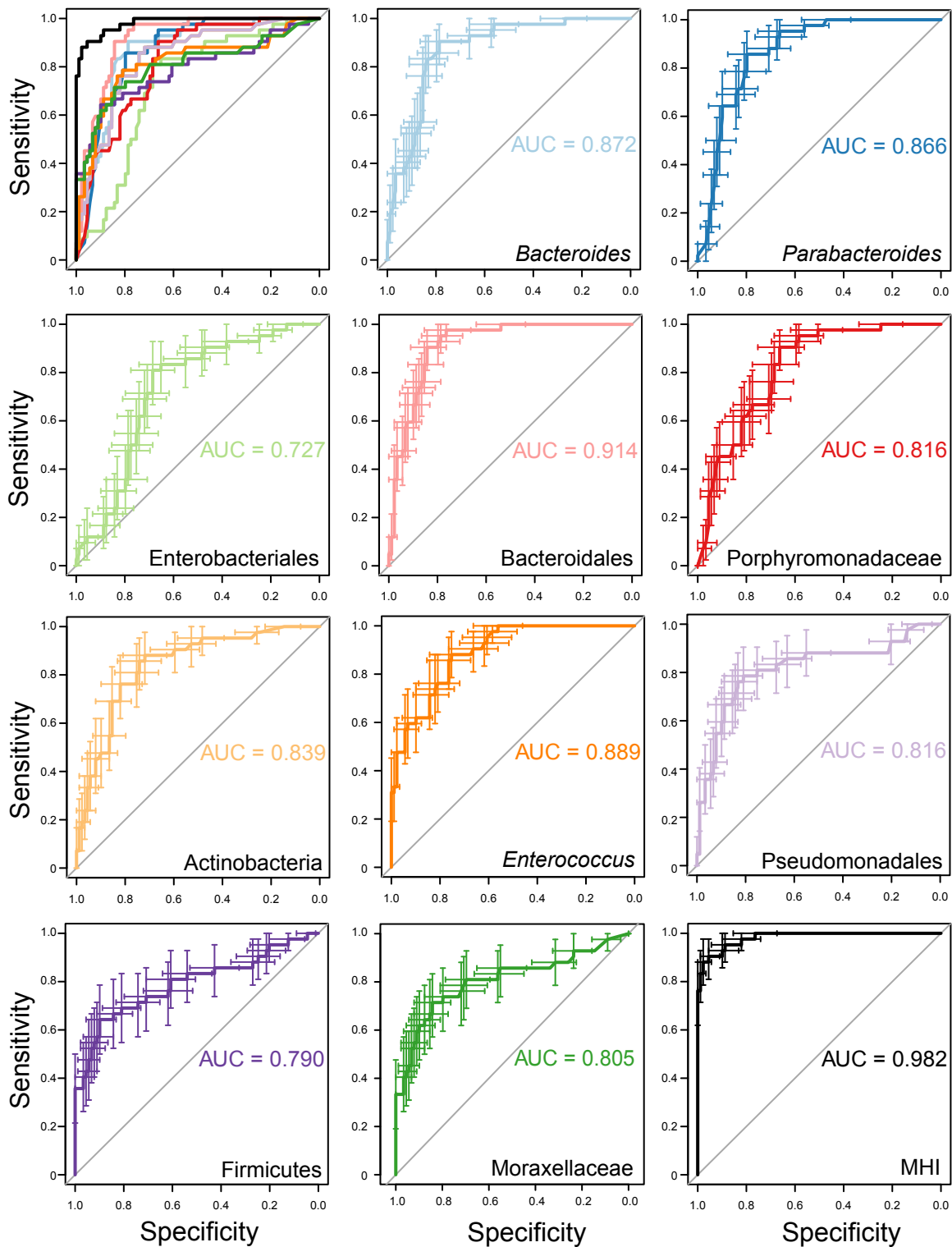

Supplement: Supplementary Figure S4 — Comparison of the ability to classify ICU-enterotypes between the ten taxonomic biomarkers and the MHI score The ROC curve with 95% CI and AUC with 95% CI were generated for the 131 fecal samples using 9999 stratified bootstrap replicates. [file mmc4.pdf]
