# Supplementary material for: Classification of the Gut Microbiota of Patients in Intensive Care Units During Development of Sepsis and Septic Shock
Source: Genomics Proteomics Bioinformatics. 2021 Feb 17;18(6):696–707. doi: 10.1016/j.gpb.2020.06.011 (PMC8377022; doi:10.1016/j.gpb.2020.06.011)
Supplement: Supplementary Figure S3 — Distributions of antibiotic use before/after ICU admissionand infection sites for samples in ICU-enterotype space The circle color and shape indicate the ICU-enterotypes and characteristics, including antibiotic type (A and B) and infection site (C). In all plots, shaded ellipses represent the 80% CI; dotted ellipses represent the 95% CI. Boxes represent the IQR between the first and third quartiles; the line inside represents the median. Whiskers denote the lowest and highest values within 1.5× IQR from the first and third quartiles, respectively. Statistical significance was tested using the Mann-Whitney-Wilcoxon test (***, P < 0.001; n.s., not significant). CI, confidence interval; IQR, interquartile range. [file mmc3.pdf]

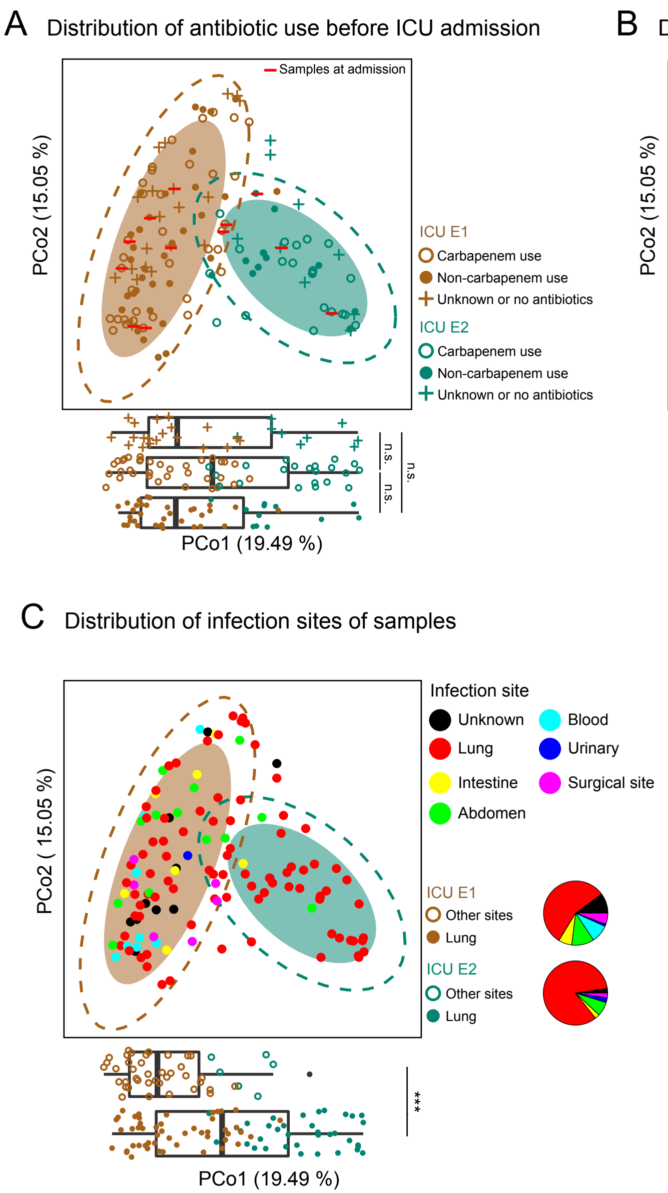

A
Distribution of antibiotic use before ICU admission
Samples at admission
PCo2 (15.05 %)
PCo1 (19.49 %)
ICU E1
Carbapenem use
Non-carbapenem use
Unknown or no antibiotics
ICU E2
Carbapenem use
Non-carbapenem use
Unknown or no antibiotics
n.s.
n.s.
n.s.
B
Distribution of antibiotic use after ICU admission
Samples at admission
PCo2 (15.05 %)
PCo1 (19.49 %)
ICU E1
Carbapenem use
Non-carbapenem use
No antibiotics
ICU E2
Carbapenem use
Non-carbapenem use
No antibiotics
n.s.
n.s.
n.s.
C
Distribution of infection sites of samples
PCo2 (15.05 %)
PCo1 (19.49 %)
Infection site
Unknown
Blood
Lung
Urinary
Intestine
Surgical site
Abdomen
ICU E1
Other sites
Lung
ICU E2
Other sites
Lung
***
